# Protection of Halophytes and Their Uses for Cultivation of Saline-Alkali Soil in China

**DOI:** 10.3390/biology10050353

**Published:** 2021-04-22

**Authors:** Lili Liu, Baoshan Wang

**Affiliations:** Shandong Provincial Key Laboratory of Plant Stress Research, College of Life Sciences, Shandong Normal University, Jinan 250014, China; 17853728922@163.com

**Keywords:** halophytes, protection, saline-alkali land, sustainable utilization

## Abstract

**Simple Summary:**

Over 800 million hectares of arable lands are affected by salinity worldwide. All crops cannot grow in saline-alkali soils due to high salt content. However, halophytes are a special category of plants that grow in saline soils. Halophytes have potential economic value as grain, vegetable, fruit, medicine, animal feed, biofuel feedstocks, and in greening and coastal protection. These also provide possible directions for the development of saline-alkali land. In addition, we are also concerned about the coordinated and sustainable development of the protection and utilization of halophytes.

**Abstract:**

Over 800 million hectares of arable lands are affected by salinity in the world. In China, saline-alkali soils account for 25% of farmland and are underutilized. One sustainable strategy to make better use of saline land is to plant halophytes, salt-tolerant plants that can survive and complete their life cycle in media containing more than 200 mM NaCl. Halophytes have potential economic value as grain, vegetable, fruit, medicine, animal feed, and biofuel feedstocks, and in greening and coastal protection. Therefore, the cultivation and protection of halophytes is very important. In the past few decades, a lot of work has been done on the protection and utilization of halophytes in saline soil improvement and development worldwide. This article focuses on the distribution of saline-alkali conditions and current measures to protect halophytes, as well as the application of halophytes in the sustainable development of saline-alkali land. This information is helpful for protection and utilization of halophytes in the sustainable development of saline land worldwide.

## 1. Area and Distribution of Saline Land

The availability of land for crop cultivation is rapidly decreasing due to industrialization, urbanization, and salinization worldwide, particularly in developing countries such as China. Salt stress is a major environmental factor limiting plant distribution, growth, and crop production [1,2]. Soil salinity affects approximately 800 million hectares of arable lands worldwide [3,4,5]. Total saline land is about 1125 million hectares in the world [6]. Furthermore, 20% of saline land is distributed in the irrigated land. Especially in the Middle East, North America, and Oceania countries, it can even reach 30% of the irrigated land [7]. The area of saline soil in Canada and the United States is 7,238,000 and 8,517,000 hectares, respectively [8]. In addition, in India, there are 7 million hectares of saline-alkali land [9]. Indeed, every minute, three hectares of arable land are degraded due to increasing soil salinity [10]. Developing strategies to make use of saline land will be crucial for addressing the problem of insufficient cropland and meeting the challenge of providing food security for the projected global population of 9.3 billion people by 2050.

**Figure 1:**
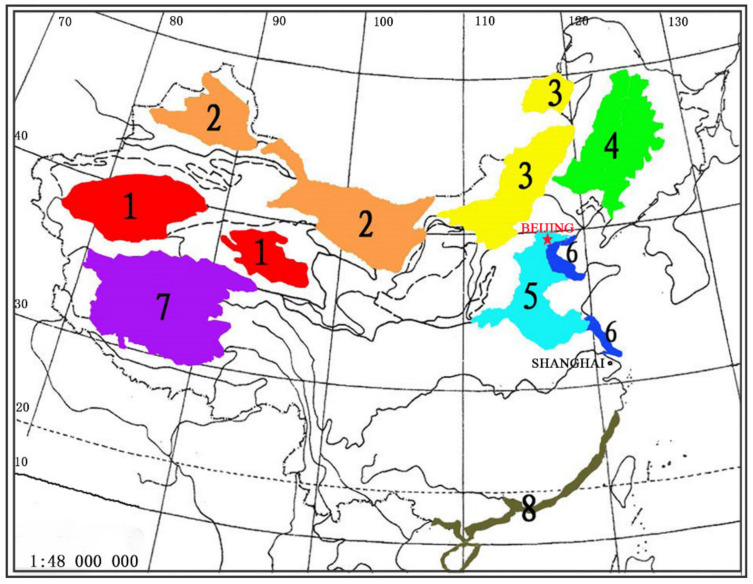
Distribution of saline soils in China. 1. Extremely arid saline soil in inland basin; 2. arid saline soil area of inland basin; 3. semi-arid saline soil area of Inner Mongolia Plateau; 4. semi-arid and semi-humid saline soil area in Northeast Plain; 5. semi-arid and semi-humid saline soil area of Huanghuaihai Plain; 6. coastal saline soil area; 7. alpine and arid saline soil area of Tibet Plateau; 8. tropical and subtropical salt marsh area.

In total, 99.13 million hectares of saline soil are mainly distributed in northern China [11]. In China, the distribution of saline-alkali land is mainly in the northern provinces of the Yangtze River due to the geographic and climatic characteristics of this area, which is divided into eight regions (Figure 1) [12,13,14]. These saline regions include the extremely arid saline soil region in the inland basin, the arid saline soil region of the inland basin, the semi-arid saline soil region of the Inner Mongolia Plateau, the semi-arid and semi-humid saline soil region in the Northeast Plain, the semi-arid and semi-humid saline soil region of the Huanghuaihai Plain, the coastal saline soil area, the alpine and arid saline soil region of the Tibet Plateau, and the tropical and subtropical salt marsh region (mangrove area). The formation of the saline soils in these regions differs. For example, in Shandong Province, the saline-alkali land is mainly distributed in the Yellow River Delta because the Yellow River brings sediment as it enters the Bohai Bay, filling the bay to create new land. Underneath the new land is seawater and as the water slowly evaporates, the land becomes saline-alkali. Saline soils are divided into three categories: coastal salt marshes, inland saline lands, and heavily irrigated soil [15]. The first two types of soil occur naturally. Plants in these lands grow in seawater (such as mangroves) or near the shores of inland lakes (such as *Kalidium foliatum* around Qinghai Lake). By contrast, other saline soils are caused by human activities. For example, in arid areas, heavily irrigated soils undergo strong evaporation. These unsustainable irrigation practices have resulted in increased salinization of agricultural lands over the past several centuries. Various strategies have been developed to improve and utilize saline-alkali land [16,17,18], such as planting halophytes for food and grazing.

## 2. Definition of a Halophyte and Types of Halophytes

Halophytes are defined as plants that can survive and complete their life cycle in media containing equal to or more than 200 mM NaCl [1]. The distinction between halophytes and non-halophytes is that in halophytes, the appropriate salinity promotes vegetative and reproductive growth [1,19,20], including salinity levels at which 99% of non-halophyte plants would die.

Halophytes are divided into three categories based on their salt-tolerance mechanism [21]: (1) Euhalophytes, such as *Suaeda salsa* L., have succulent leaves and stems that compartmentalize Na^+^ and Cl^−^ into the vacuoles. (2) Pseudohalophytes or salt excluders, such as the *Phragmites australis*, are able to prevent salt from entering the shoot so the leaf Na^+^ content per dry biomass is much lower than that of the root. Little is known about the salt restriction mechanism of salt excluders [22], but it could be due to the specific apoplastic barriers in the endodermis of the roots [23]. (3) Recretohalophytes, such as *Limonium bicolor* and *Chenopodium quinoa*, avoid salt damage via unique structures including salt glands and salt bladders that actively excrete ions out of the plant [3].

Halophyte species account for 1~2% of the world’s land plants [11,15]. There are approximately 235,000 plant species in the world, of which salt-tolerant and halophytes account for approximately 26,000 species. Among the 2891 species of higher plants, there are 25 species of halophytes and 150 species of salt-tolerant plants [8]. Halophytes and their distribution in most areas of saline land in China have been surveyed since 1999. We used Halophytes database, eHALOPH (https://www.sussex.ac.uk/affiliates/halophytes/, accessed on 5 March 2020) to find that there are 419 halophyte species in China, belonging to 198 genera and 66 families (Table 1) [11,24,25].

## 3. Protection of Halophytes in China

At present, only a few halophytes are cultivated and used for economic purposes; most halophytes are still only found in the wild. However, studies of halophytes are an active area of research, as these species are promising sources of salt-tolerance genes that can be used to improve the salt tolerance of crops and as potential resources for vegetables, fruit trees, energy plants, greening plants, and so on. Some halophytes can be used for food, biofuels, greening, and medicine. The deterioration of saline land makes it urgent for us to protect halophyte resources.

Different laws and resources have been introduced to protect halophytes in different countries. In China, legislation, establishment of National Natural Reserves focusing on halophytes, and the establishment of the Halophyte Germplasm Resource Bank are helping to protect halophytes. According to Article 9 of the Regulations on the Protection of Wild Plants of the People’s Republic of China, revised in 2017, the state protects wild plants and their habitats. It is forbidden for any group or individual to collect wild plants or destroy their environment. According to Article 11, in the natural distribution areas of nationally protected wild plant species and local key protected wild plant species, people shall act in accordance with relevant laws and administrative regulations. The List of Rare and Endangered Protected Plants in China, published by the State Environmental Protection Agency and the Chinese Academy of Sciences, lists 18 species of endangered halophytes in China. Among them, salt birch (*Betula halophila* Ching ex P. C. Li), wild soybean (*Glycine soja* Sieb. et Zucc.), red grove plum (*Lumnitzera littorea* (Jack) Voigt), and others are considered to be secondary key protected plants, which refers to endangered or economically, scientifically, culturally, and genetically valuable species.

The Chinese Halophyte Germplasm Resource Bank at Shandong Normal University includes 383 species of halophytes, along with halophyte seeds and electronic data. This provides a great resource for the study of the salt-tolerance mechanism of halophytes, salt-tolerance breeding, and resource protection.

Nature reserves can protect halophytes in situ, protecting not only the species, but also the natural habitat in which they grow. For example, the Yellow River Delta Nature Reserve, Mangrove Forest Nature Reserve, and Aibi Lake Wetland Nature Reserves are important locations for the protection of halophytes in China.

## 4. Utilization of Halophytes

**Figure 2:**
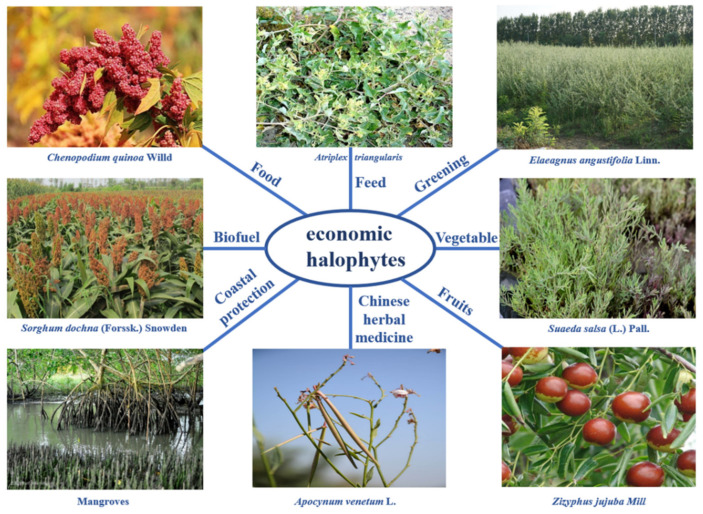
Representative species of halophytes applied in China.

Halophytes are precious natural resources and have potential economic value [26] as grain, vegetable, fruit, medicine, animal feed, and biofuel feedstocks, and in greening and coastal protection. Some halophytes, such as limonium, can improve the salt tolerance of non-halophytes. Halophytes have a powerful antioxidant system, which contains highly active natural antioxidants. They will have great development prospects for human health in the future [27]. Many halophytes can also grow on toxic metal soils and produce yields, and under the same environment they have stronger environmental adaptability than non-halophytes, which provides a new idea for the treatment of environmental pollution [28]. The main halophytes used in China are shown in Table 2 and Figure 2 [11,29,30,31,32,33].

### 4.1. Halophytes as Model Plants for Studying the Mechanisms of Plant Salt Resistance

Halophytes have evolved unique strategies to adapt to salinity. *L. bicolor* is a recretohalophyte that possesses salt glands that directly secrete excess ions out of the plant to avoid salt damage [10,34,35]. *L. bicolor* is a promising model plant for studying the molecular mechanisms of salt gland development and salt secretion because of the availability of established regeneration systems, genetic transformation systems, mutant libraries, transcriptome data, and knowledge of key genes related to development and the process of salt secretion [3,19,20,36,37,38,39,40,41,42,43,44]. In addition, genome sequencing of *L. bicolor* has also been completed and the sequence will be released soon. Another halophyte model plant is *S. salsa*, which has succulent leaves and stems and can grow in high salinity conditions, even in intertidal zones [45]. In short, halophytes have great potential as genetic resources for salt-tolerance breeding, and more attention must be paid to the detailed molecular mechanisms of salt tolerance in halophytes.

### 4.2. Halophytes Used for Ecological Protection and Sustainable Development of Regional Economies

Mangroves are important halophyte plants in tropical and subtropical tidal flats such as Guangdong, Guangxi, Fujian, and Hainan, and are of great importance for ecological maintenance, purification, and marine aquaculture [46]. There are 38 mangrove species in China [29,47]. However, since the 1950s, mangrove populations have been severely damaged and nearly 50% of mangrove populations in China have disappeared due to natural factors such as the sea level rise due to climate warming and human disturbance [48,49]. The rapid decrease of mangrove populations has led to increased tidal flat pollution in coastal areas such as Fujian, Guangdong, and Guangxi. Fisheries resources are increasingly becoming scarce, and the environment has been severely damaged. Therefore, the protection of mangrove habitats has an important role in sustaining the regional economy [48,50,51,52]. Furthermore, studies have shown that the growth rate of *Sesuvium portulacastrum* increased in a salt environment, and that it has a higher ability to accumulate salt, thereby improving the soil [53]. Studies have shown that halophytes promote the growth of rhizobacteria which can stimulate plant growth and increase the salt tolerance of non-saline crops [54]. At present, China’s government is formulating plans to increase investment in scientific research and ecological restoration to restore and protect mangrove habitats.

### 4.3. Halophytes Used for Greening and Ecological Reconstruction in Coastal Cities

There are only a few species of trees (*Elaeagnus angustifolia* and *Tamarix chinensis*) in northern coastal areas such as Dongying, Tianjin, and Cangzhou, which is a major factor restricting urban greening and ecological reconstruction in these regions. At present, the greening of coastal cities in northern China is carried out by soil replacement and water conservation projects. Although effective, the cost of construction and maintenance is huge and unsustainable. The words “one year green, two years yellow, and three years dead” are a portrayal of the fate of urban plantings in coastal saline-alkali areas of China. Since 1994, three halophyte gardens have been constructed in the Yellow River Delta where 264 halophyte species were introduced. These halophytes can all grow and complete their life cycle in this environment, forming a unique natural landscape, and some of them are widely used as tree species for landscaping and ecological restoration [55,56,57].

### 4.4. Halophytes Used for Forage

Many of the saline-alkali areas in China have a small human population and underdeveloped industrialization, which is very suitable for large-scale animal husbandry. However, most of the forage grass in these regions is imported or purchased from Gansu, Ningxia, and Australia, and the cost is very high [58,59,60]. Locally grown forage could greatly reduce costs and promote the development of animal farming. Although forages grow slowly on saline-alkali soils, the forage produced from saline land improves the quality, tenderness, and nutritional content of meat from cattle and sheep [61,62]. It is true that better beef and mutton are produced in saline-alkali regions such as Xinjiang and Ningxia in China [63,64]. To identify salt-tolerant forage species, 308 sorghum (*Sorghum bicolor*) varieties were tested for salt tolerance, and more than 100 salt-tolerant varieties were planted on saline land of Dongying Qingtuo Farm [65]. About 110 tons of fresh forage was produced per hectare. Our research group is cooperating with companies and will transfer cultivation techniques to large dairy farms or sheep farms to generate economic and social benefits in saline land in China.

### 4.5. Halophytes Used for Biofuel

In China, energy is now mainly dependent on the import of crude oil, which accounts for about 60% of the energy produced in China. Crude oil, as well as gas and coal, are non-renewable resources. These fossil fuels will be used up in the next few decades [66]. China attaches great importance to the investment and development of renewable energy sources such as solar energy, wind energy, hydropower, nuclear energy, and biofuels. However, these forms of energy are all limited by geographical location and natural conditions, except biomass energy [15,67]. Therefore, China has prioritized the development of biomass energy. However, land for the cultivation of biofuel crops is limited, and the development of biomass energy must use marginal land such as salt-alkali land. Sweet sorghum and *Manihot esculenta* are widely planted in saline soil. Sweet sorghum, which produces high-sugar stalks, can produce about 100 tons of fresh stalks per hectare, which can generate about 5 tons of bioethanol.

### 4.6. Halophytes Used for Medicine

Many halophytes have medicinal value in traditional medicine. Some medicinal materials from saline land have higher contents of effective ingredients than those from non-saline-alkali land. In rural areas, many halophyte medicinal plants can be used to treat diseases and bacterial and fungal infections. Although there is no theoretical basis at present, many halophytes are still widely used as medicine [26]. *Mesembryanthemum edule* L. can be used as a treatment for sinusitis, diarrhea, infantile eczema, and tuberculosis [68]. *Eryngium maritimum* L is a halophyte that grows along the Atlantic and Mediterranean coasts. It can be made into a variety of drugs, such as sweat medicine, bladder medicine, aphrodisiac, and expectorant [69]. *Cakile maritima* L. can not only treat diuresis, but also treat colitis and clean the phlegm in the lungs [70]. In addition, many halophytes are used in Chinese herbal medicines. Many valuable Chinese herbal medicine resources, such as *Apocynum venetum*, *Glycyrrhiza uralensis* Fisch, and *Limonium sinense* (Girard) Kuntze, grow in saline-alkali soils [71,72]. If these halophytes can be planted in saline land on a large scale, this could benefit the herbal medicine industry in saline land areas in the world.

### 4.7. Halophytes Grown for Vegetables in Seawater and Saline-Alkali Land

Many halophytes can be used as vegetables for human consumption [72]. Seawater vegetables are rich in protein, vitamins, and a variety of essential trace elements [73]. *S. salsa* grown on saline land, even in coastal areas, is widely used as a vegetable. *S. salsa* is rich in betaine, vitamins, and dietary fiber as well as salts, polysaccharides, flavones, and polyphenols [45,74]. Sea asparagus (*Salicornia bigelovii*,) has been widely planted in coastal areas of subtropical or tropical regions such as Hainan [75]. The content of trace elements and amino acids in sea asparagus is much higher than that of ordinary vegetables, and its seed protein content can reach 42.57%, which is higher than that of soybean (*Glycine max*, 35.48%) and peanut (*Arachis hypogaea*, 28%). At present, the market prices for soybeans and peanuts are 5 and 10 RMB per kilogram, respectively, but for ice plant, the market price is 26–36 RMB per kilogram. Ice plant (*Mesembryanthemum crystalinum* L.) is native to Madagascar in South Africa. Ice plant is a facultative crassulacean acid metabolism plant, induced by salinity or drought. Therefore, it can be planted in saline soils [76]. At present, ice plant has become a valuable sea vegetable in China.

### 4.8. Halophytes Used as Fruits

Several halophyte fruit trees are found in saline-alkali regions [77]. In Zhanhua, the Yellow River Delta, winter jujube (*Ziziphus jujuba* cv. Dongzao) is a well-known fruit. Interestingly, only the fruit of winter jujube growing in saline-alkali land is crisp and sweet. *Nitraria tangutorum* Bor. grows in saline-alkali land such as Xinjiang, Ningxia, and Gansu, and its fruit is very nutritious. It contains 19 kinds of amino acids, including lysine, leucine, threonine, and five other essential amino acids required by the human body.

## 5. Perspectives

Many legislations and National Natural Reserves have been made to protect halophyte in different countries of the world. However, more attention must be paid to the protection of halophytes, as their native habitats are increasingly threatened by human activity and the populations of some halophytes are decreasing. As one step in conserving these species, more National Natural Reserves should be constructed to preserve the species and their native habitats. Establishment of gardens where different halophytes can be introduced, propagated, and protected will also help preserve this important natural resource for scientific research, agriculture, and ecological uses. More cooperative works need to construct more halophyte germplasm resource banks in the world to collect and conserve most halophyte species. For halophyte utilization, the sustainable development of halophytes is an indispensable prerequisite in the future to avoid overexploitation.

## 6. Conclusions

Halophytes, as a kind of plant with various economic applications, provide us with a new direction in the development and utilization of saline-alkali soils. Using biological methods for saline-alkali utilization can not only have good economic benefits, but can also be green and sustainable development. However, we still have to be cautious about the use of halophytes. We must protect the rare and endangered halophytes, prevent excessive exploitation and utilization, and formulate relevant laws for the protection and utilization of halophytes, so as to truly achieve coordinated development between protection and utilization.

## Figures and Tables

**Table 1:** The number of the family, genus, and species of halophytes in China.

Family	Genus	Species	Family	Genus	Species
Acanthaceae	1	2	Malvaceae	3	5
Acrosti chaceae	1	2	Meliaceae	1	1
Aizoaceae	2	2	Myoporaceae	1	1
Amaranthaceae	2	2	Myrsinaceae	1	1
Apocynaceae	3	4	Najadaceae	2	3
Asdepiadaceae	3	4	Olacaeae	1	1
Betulaceae	1	1	Onagraceae	1	1
Bignoniaceae	1	1	Orobanchaceae	2	3
Boraginaceae	8	10	Palmae	1	1
Caryophyllaceae	1	1	Pandanaceae	1	1
Chenopodiaceae	17	72	Plantaginaceae	1	4
Combretaceae	2	3	Plumbaginaceae	1	11
Commelinaceae	1	1	Poaceae	21	44
Compositae	20	44	Polygonaceae	2	10
Conoolvulaceae	3	9	Potamogetonaceae	6	13
Cruciferae	4	9	Primulaceae	2	2
Cyperaceae	7	16	Ranunculaceae	1	5
Dryopteridaceae	1	1	Restionaceae	1	1
Elaeagnaceae	1	1	Rhizophoraceae	4	9
Euphorbiaceae	2	3	Rosaceae	3	3
Frankeniaceae	1	1	Rubiaceae	1	1
Goodeni aceae	1	2	Rutaceae	1	1
Guttiferae	1	1	Salicaceae	1	2
Hernandiaceae	1	1	Sapindaceae	2	2
Hydrocharitaceae	3	5	Scrophulariaceae	4	4
Iridaceae	1	3	Simaroubaceae	1	1
Juncaginaceae	1	3	Solanaceae	1	4
Labiatae	3	5	Sonneratiaceae	1	3
Lecythidaceae	1	2	Sterculiaceae	1	1
Leguminosae	18	33	Tamaricaceae	2	15
Liliaceae	1	1	Umbelliferae	6	7
Loganiaceae	1	1	Verbenaceae	3	3
Lythraceae	1	1	Zygophyllaceae	3	8

**Table 2:** Types and representative species of the applied halophytes in China.

Use	Species	Distribution	Application
Food	*Chenopodium quinoa* Willd. (Quinoa)	Quinoa is native to the Andes in South America. It has been cultivated on a small scale in Tibet, Shaanxi, Shanxi, Qinghai, Sichuan, and Zhejiang.	Quinoa seeds are edible and highly nutritious, including minerals and vitamins; moreover, their protein content is more than twice that of rice, and quinoa contains lysine, which is lacking in many grains.
Chinese herbal medicine	*Apocynum venetum* L.	*A. venetum* grows mainly in saline-alkali wastelands, the edges of deserts, riverbanks, alluvial plains.	*A. venetum* leaves can be used to treat hypertension, dizziness, insomnia, neurasthenia, and heart failure, and delay aging.
Feed	*Atriplex triangularis*	*A. triangularis* is widely distributed in saline land and used as forage. It can be irrigated with seawater and has strong salt tolerance.	*A. triangularis* has 1.5 times the vitamin C content of spinach, and is rich in many essential trace elements. The stems and leaves are rich in nutrients, and are high-quality feed for cattle, sheep, and horses.
Greening	*Elaeagnus angustifolia* Linn.	*E. angustifolia* is distributed in plains, river beaches, and saline soil.	*E. angustifolia* has a beautiful tree shape, which can be used in urban areas and to protect against wind, dust, and noise.
Vegetable	*Suaeda salsa* (L.) Pall.	*S. salsa* is mainly distributed in Qinghai, Xinjiang, Shandong, Jiangsu, and other coastal areas. The young leaves are edible and rich in the antioxidant betacyanin and trace elements.	*S. salsa* leaves have a good taste and are rich in vitamins and dietary fiber.
Biofuel	*Sorghum dochna* (Forssk.) Snowden (Sweet sorghum)	Sweet sorghum is salt-tolerant and can grow in saline soils containing 0.2–0.6% salt.	*S. dochna* has high biomass and sugar content. As energy grasses, they can be converted into solid, liquid, or gaseous biofuels.
Fruit	*Zizyphus jujuba Mill* (Winter jujube)	Winter jujube is a native species and mainly distributed in saline soil of the Bohai Bay area at the border of Hebei and Shandong Province.	Winter jujube is a well-known fruit and is rich in vitamins, calcium, iron, zinc, and essential amino acids such as aspartic acid, threonine, and serine.
Coastal protection	*Acanthus ilicifolius, Rhizophora mangle, Barringtonia racemose* (Mangroves)	Mangroves are a unique woody plant community in tropical and subtropical intertidal zones and are mainly distributed along the coasts of Guangxi, Guangdong, and Taiwan.	Mangroves play an important role in reducing coastal damage from waves, protecting beaches, purifying seawater, maintaining coastal ecological balance, and marine aquaculture.

## Data Availability

Data are contained within the article and Halophytes database, eHALOPH (https://www.sussex.ac.uk/affiliates/halophytes/, accessed on 18 April 2021).
